# Investment Success in Public Health: An Analysis of the Cost-Effectiveness and Cost-Benefit of the Global Programme to Eliminate Lymphatic Filariasis

**DOI:** 10.1093/cid/ciw835

**Published:** 2016-12-10

**Authors:** Hugo C. Turner, Alison A. Bettis, Brian K. Chu, Deborah A. McFarland, Pamela J. Hooper, Sunny D. Mante, Christopher Fitzpatrick, Mark H. Bradley

**Affiliations:** 1 London Centre for Neglected Tropical Disease Research, and; 2 Department of Infectious Disease Epidemiology, School of Public Health, Faculty of Medicine, St Marys Campus, Imperial College London, United Kingdom;; 3 Oxford University Clinical Research Unit, Wellcome Trust Major Overseas Programme, Ho Chi Minh City, Vietnam;; 4 Centre for Tropical Medicine and Global Health, Nuffield Department of Medicine, University of Oxford, United Kingdom;; 5 Neglected Tropical Diseases Support Center, Task Force for Global Health, Decatur, and; 6 Rollins School of Public Health, Emory University, Atlanta, Georgia;; 7 Urology Unit, 37 Military Hospital, Korle-Bu, Accra, Ghana;; 8 Department of Control of Neglected Tropical Diseases, World Health Organization, Geneva, Switzerland; and; 9 Global Health Programs, GlaxoSmithKline, London, United Kingdom

**Keywords:** lymphatic filariasis, economic evaluation, preventive chemotherapy, cost-benefit, hydrocelectomy.

## Abstract

**Background.:**

It has been estimated that $154 million per year will be required during 2015–2020 to continue the Global Programme to Eliminate Lymphatic Filariasis (GPELF). In light of this, it is important to understand the program’s current value. Here, we evaluate the cost-effectiveness and cost-benefit of the preventive chemotherapy that was provided under the GPELF between 2000 and 2014. In addition, we also investigate the potential cost-effectiveness of hydrocele surgery.

**Methods.:**

Our economic evaluation of preventive chemotherapy was based on previously published health and economic impact estimates (between 2000 and 2014). The delivery costs of treatment were estimated using a model developed by the World Health Organization. We also developed a model to investigate the number of disability-adjusted life years (DALYs) averted by a hydrocelectomy and identified the cost threshold under which it would be considered cost-effective.

**Results.:**

The projected cost-effectiveness and cost-benefit of preventive chemotherapy were very promising, and this was robust over a wide range of costs and assumptions. When the economic value of the donated drugs was not included, the GPELF would be classed as highly cost-effective. We projected that a typical hydrocelectomy would be classed as highly cost-effective if the surgery cost less than $66 and cost-effective if less than $398 (based on the World Bank’s cost-effectiveness thresholds for low income countries).

**Conclusions.:**

Both the preventive chemotherapy and hydrocele surgeries provided under the GPELF are incredibly cost-effective and offer a very good investment in public health.

## Summary

We found that both the preventive chemotherapy and hydrocele surgery provided under the Global Programme to Eliminate Lymphatic Filariasis (GPELF) are cost-effective and offer a very good investment in public health. These results clearly demonstrate the value and importance of continued investment in the GPELF and show that continued funding is justified and highly cost-effective.

Before widespread control, approximately 120 million people worldwide were infected with lymphatic filariasis (LF), with 40 million suffering from overt clinical disease [1, 2]. Though infection is often asymptomatic, clinical disease occurs in around one-third of infected individuals and can manifest as hydrocele, lymphedema, and acute adenolymphangitis episodes. Clinical disease can be debilitating and reduces economic productivity as well as limiting educational and employment opportunities. Those suffering from physical disfigurement often experience stigmatization and discrimination [3].

In 2000, the World Health Organization (WHO) established the Global Programme to Eliminate Lymphatic Filariasis (GPELF), with the goal of eliminating the disease as a public health problem by 2020 [4]. The program has the following 2 parallel goals: to use community-wide preventive chemotherapy to interrupt transmission and to provide access to a basic package of care to every affected person in endemic areas in order to manage morbidity and prevent disability. These goals are supported with the WHO’s 2020 neglected tropical disease (NTD) Road Map [5].

The GPELF has been incredibly successful, delivering more than 5.6 billion preventive chemotherapy treatments between 2000 and 2014 (Supplementary Table S1). However, in order to achieve the WHO’s 2020 Road Map target, the 21 countries with incomplete geographical coverage (as well as the 11 countries that have yet to start drug distribution) will need to scale up preventive chemotherapy fully. Furthermore, only 24 (33%) endemic countries have established morbidity management and disability prevention programs [6].

It has been estimated that $154 million ($105–$208 million) per year will be required during 2015–2020 to continue the GPELF [7]. In light of this, it is important to understand the program’s current value. Here, we address the program’s value by evaluating the cost-effectiveness and cost-benefit of the preventive chemotherapy that was provided under the GPELF between 2000 and 2014. In addition, we perform the first analysis to investigate the potential cost-effectiveness of hydrocele surgery.

## METHODS

### 

#### Effectiveness of Preventive Chemotherapy

Turner et al [8] estimated the health and economic impact of the preventive chemotherapy provided by GPELF on those treated between 2000 and 2014 (Supplementary Table S2). A summary of the baseline model assumptions is shown in Supplementary Figure 1. It was estimated that due to the first 15 years of the GPELF, 36 million chronic cases and 115 million disability-adjusted life years (DALYs) (Box 1) would be averted over the lifetime of the treated population (Supplementary Table S2)

**Box 1 Glossary**

**Benefit-cost ratio:** The ratio of the monetary benefits of a program relative to its costs.

**Cost-effectiveness ratio:** A statistic used to summarize the cost-effectiveness of a healthcare intervention. It is defined as the cost of an intervention divided by its health effect.

**Disability-adjusted life years (DALYs):** A measure of disease burden calculated as the sum of the years of life lost due to premature mortality and the years lost due to disability. The number of years lost due to disability is calculated using a disability weight factor (between 0 and 1) that reflects the severity of the disease. One DALY can be thought of as 1 year of “healthy” life lost.

**Direct costs:** The value of all goods, services, and other resources consumed in providing healthcare. These can be split into 2 types: the costs borne by the health system (eg, personnel, hospital services,) and the costs borne by patients/the community (eg, the costs patients pay for transport to the hospital and for medication).

**Discounting/discount rate:** The process of adjusting future costs and outcomes to a “present value.” The discount rate determines the strength of the time preference.

**Economic costs:** The full value of all resources used (including for donated items for which no financial transaction has taken place). For example, the time devoted to mass drug administration (MDA) by Ministries of Health staff has an economic cost but not a financial cost. These are important when considering issues related to the sustainability and replicability of interventions.

**Financial costs:** The accounting cost (ie, actual amount paid) for a good or service.

**Indirect costs:** Clinical lymphatic filariasis is debilitating, and it reduces productivity (ie, number of work hours). The economic value of this productivity loss is an indirect cost. This is not necessarily an out-of-pocket payment (financial cost) but an opportunity cost of productive time lost.

**Perspective:** The study perspective is the viewpoint from which the intervention’s costs and consequences are evaluated.

**Mass drug administration (MDA):** MA means of delivering treatment based on the principles of preventive chemotherapy, where populations or sub-populations are offered treatment without individual diagnosis.

No projections were made for the expansion of the mass drug administration (MDA) programs after 2014 or their resulting benefits [8]. The economic benefits associated with prevention of this clinical disease was then analyzed in the context of prevented medical expenses incurred by LF clinical patients, potential income loss through lost labor, and prevented costs to the health system to care for those affected—aggregating the benefits over the lifetime of the benefit cohort (Supplementary Table S2). A summary of the sensitivity analysis performed on the effectiveness of preventive chemotherapy is shown in Supplementary Table S3.

For further details regarding the effectiveness calculations, see Turner et al [8].

#### Costs of Preventive Chemotherapy

We considered both the financial costs (ie, the actual cash disbursements for a program) and economic costs (ie, the value of all resources used in the program, including donated resources) incurred for the preventive chemotherapy provided under the GPELF between 2000–2014. This includes both the cost of the drugs/their economic value and the costs associated with their delivery. The analysis stratified the costs in the following 3 ways: financial costs only; economic costs, excluding the value of donated drugs; and economic costs, including the value of donated drugs (Table 1). The costs are expressed in US 2014 dollars.

**Table 1.** Summary of Drug and Treatment Costs

**Drug**	**Average Cost/Economic Value per Treatment** ^a^
DEC	$0.044
ALB	$0.052
IVM	$4.635
**Treatment delivery cost type**	**Average Cost Per Treatment (95% Confidence Interval**)
Financial costs	$0.46 ($0.21–$0.76)
Economic costs excluding DDV	$0.56 ($0.25–$0.94)
Economic costs including DDV (overall average of the Global Programme to Eliminate Lymphatic Filariasis)	$1.32 ($1.00–$1.69)
Economic costs including DDV (IVM and ALB regimen)	$5.25 ($4.93–$5.62)
Economic costs including DDV (DEC and ALB regimen)	$0.66 ($0.34–$1.03)

Prices were adjusted to 2014 US dollars [11]. When estimating the delivery costs, we used the model parameterization [9,10] relating to the use of paid health workers and not community volunteers (resulting in a higher unit delivery cost). Further description is provided in Supplementary Tables S4 and S5. The total cost of the program for 2000–2014 was estimated by multiplying the relevant unit costs by the numbers treated [12] for each year over this time period.

Abbreviations: ALB, albendazole; DDV, donated drugs value; DEC, diethylcarbamazine; IVM, ivermectin.

^a^Includes a wastage factor of 10%.

A summary of the drug costs/economic value is provided in Table 1. The delivery costs of MDA were estimated using a recently developed web-based regression model developed by WHO (Table 1) [9, 10]. Further description is provided in Supplementary Tables S4 and S5.

### 

#### Effectiveness of Hydrocele Surgery

Hydrocelectomy is a surgical procedure that drains excess intrascrotal fluid (ie, hydrocele) and inverts or removes the tunica vaginalis (the tissue that produces this fluid) to prevent hydrocele from recurring.

For men living with a hydrocele, the surgery ensures they are able to return to their daily activities, continue to contribute to the prosperity of their family and community, and resume a normal life. We performed an economic evaluation of a hydrocelectomy to give an indication of its cost-effectiveness and how sensitive the estimates are to a range of different assumptions and parameters (outlined in Supplementary Table S6).

The number of DALYs averted from a hydrocelectomy was derived from the assumed average age at surgery, the average life expectancy in LF endemic areas, and the relevant LF DALY weight (Supplementary Table S6). Based on data provided by Kiddoo et al [13], a typical hydrocelectomy was assumed to have a success rate of 87% (with the remaining 13% either failing to reverse the condition or resulting in the patient having severe adverse outcomes). We assumed that a successful hydrocelectomy alleviates 90% of the morbidity associated with living with hydrocele (Supplementary Table S6). A 1-month lag between the time of surgery and any post-surgical health benefit was assumed and varied in the sensitivity analysis (Supplementary Table S6).

#### Costs of Hydrocele Surgery

A hydrocelectomy is not very expensive, with reported costs ranging from $80 to $360 [7]. However, no comprehensive costing studies have been published. Due to this lack of data, we considered a wide range of costs and investigated the conditions under which hydrocelectomy would be classed as cost-effective in a low income country. It should also be noted that patients can incur additional costs for a hydrocelectomy [14]. Based on the research of Ahorlu et al [14], we assumed that a hydrocelectomy would cost an average patient $37 in transportation and lost wages (outlined in Supplementary Table S7).

### Outcomes

We performed a cost-effectiveness analysis of the preventive chemotherapy provided under GPELF between 2000–2014 and of a hydrocelectomy—calculating the cost per DALY averted (the cost-effectiveness ratio). The counterfactual for both sets of analyses was no intervention. If the cost-effectiveness ratio was less than $246 per DALY averted, the intervention was considered cost-effective (and highly cost-effective if less than $41 per DALY averted). This is based on the thresholds established by the World Bank for low income countries [15] (inflated to their 2014 equivalent [11]). It should be noted that we used these thresholds in order to be conservative and higher thresholds would be applicable for middle-income countries. We also calculated the benefit-cost ratio of the preventive chemotherapy delivered between 2000–2014, which is the monetary gain realized by an intervention divided by its cost. The outcome metrics and health economic terms are defined in Box 1. As noted by Turner et al [8], we used the base year of 2014 and discounted the effects at 3% per year from 2014 onward.

When investigating the cost-effectiveness of preventive chemotherapy, the perspective for the intervention’s costs was that of the healthcare provider since the costs for individuals receiving the intervention are negligible. However, for the analysis of hydrocelectomy, we also used a wider societal perspective for the surgery’s cost, which includes costs incurred by both the health system and individual patients. The projected economic benefits due to prevented potential income loss (through lost labor) (Supplementary Table S3) were only considered in the cost-benefit analysis.

## RESULTS

### Cost-Effectiveness and Cost-Benefit of Preventive Chemotherapy

We found that regardless of the cost type we used, the preventive chemotherapy provided under the GPELF between 2000–2014 would be classed as cost-effective (defined as less then $246 per DALY averted). Furthermore, when the economic value of the donated drugs was not included, the preventive chemotherapy would be classed as highly cost- effective (defined as less then $41 per DALY averted). The results for the different cost types are outlined in Table 2, with the range being based on the confidence intervals surrounding the treatment delivery costs. When the economic costs were used (excluding the value of donated drugs), the projected benefit-cost ratio was 30 (18–63). Inclusion of the economic value of the donated drugs in the analysis decreased the projected cost-effectiveness, but it would still be classed as cost-effective and had a benefit-cost ratio of 14 (11–18) (Table 2). The change in cost-effectiveness was most substantial for the countries that use the ivermectin and albendazole regimen due to the higher reported economic value of ivermectin (Table 1 and Table 2).

**Table 2.** Cost-Effectiveness Ratios and Benefit-Cost Ratios of Preventive Chemotherapy

**Costs Type**	**Cost per Disability-Adjusted Life Years Averted ($**)^**a**^	**Benefit-Cost Ratio** ^**a**^
Financial costs	24 (12–39)	36 (23–74)
Economic costs excluding DDV	29 (14–48)	30 (18–63)
Economic costs including DDV (overall average of Global Programme to Eliminate Lymphatic Filariasis)	64 (49–83)^b^	14 (11–18)

Results are in 2014 US dollars.

Abbreviation: DDV, donated drugs value.

^**a**^Range based on the predicted 95% confidence intervals for the treatment delivery costs.

bStratified by drug regimen the cost-effectiveness ratios were 258 (243–276) for the ivermectin and albendazole regimen and 34 (18–52) for the diethylcarbamazine and albendazole regimen.

#### Sensitivity Analysis

**Figure 1.**
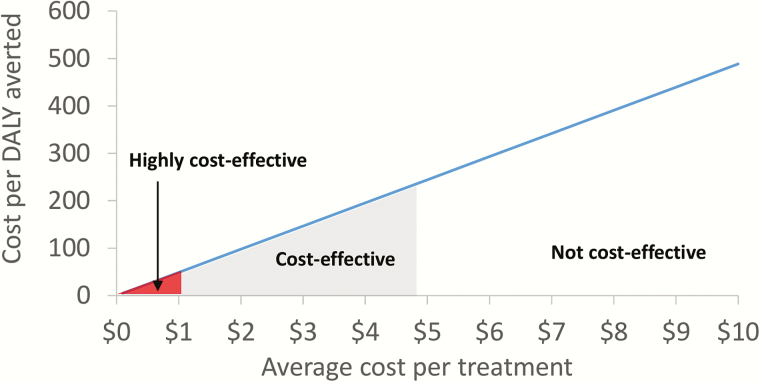
Sensitivity of the cost-effectiveness of preventive chemotherapy to the assumed average treatment cost. If the cost-effectiveness ratio was less than $246 per disability-adjusted life years (DALY) averted, it was considered cost-effective and highly cost-effective if less than $41 per DALY. This is based on the thresholds established by the World Bank for low income countries [15] (inflated to their 2014 equivalent [11]). Abbreviation: DALY, disability-adjusted life years.

**Figure 2.**
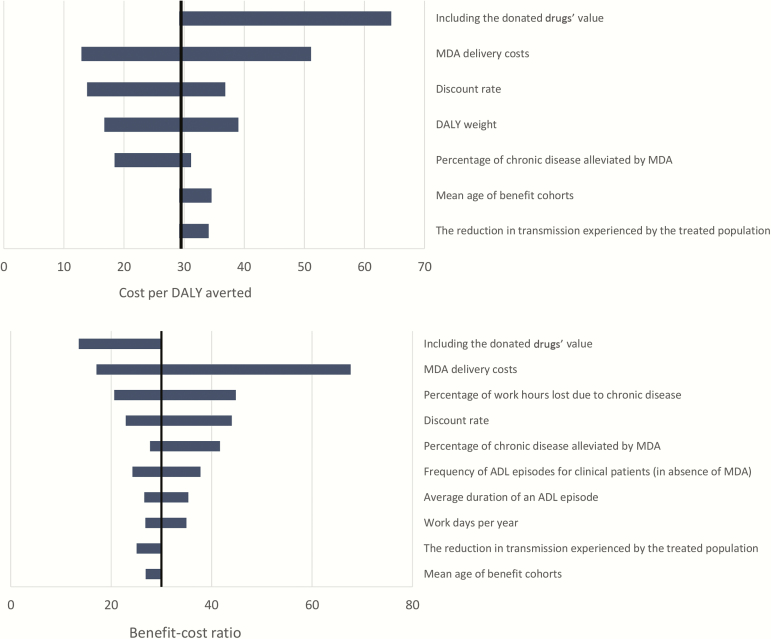
Impact of the sensitivity analysis on the estimated cost-effectiveness and cost-benefit of the preventive chemotherapy provided under the Global Programme to Eliminate Lymphatic Filariasis (2000–2014). The parameter ranges investigated are shown in Supplementary Table S3 and in Table 1. Baseline results assume the economic delivery costs (excluding the donated drugs value). For transparency, parameters that have less than a 10% impact on the outcome are shown in Supplementary Figure S2. Abbreviations: ADL, acute adenolymphangitis; DALY, disability-adjusted life years; MDA, mass drug administration.

The projected cost-effectiveness and cost-benefit of the preventive chemotherapy provided under the GPELF were sensitive to the assumed treatment costs. However, our findings were robust even when we used costs beyond the upper 95% confidence interval of the estimated treatment delivery costs (Figures 1 and 2 and Table 2).

The sensitivity of the cost-effectiveness/cost-benefit results for preventive chemotherapy is illustrated in Figure 2, which illustrates the change in the cost-effectiveness/cost-benefit affecting each model parameter listed in Supplementary Table S3 and in Table 2. The cost-effectiveness was sensitive to the assumed treatment cost and disability weight but overall was found to be relatively robust. The benefit-cost ratio was most sensitive to the assumed treatment cost and the percentage of work hours lost due to chronic disease. When a higher discount rate (6%) was used, the cost-effectiveness and cost-benefit decreased but were still promising.

### Cost-Effectiveness of Hydrocele Surgery

**Figure 3.**
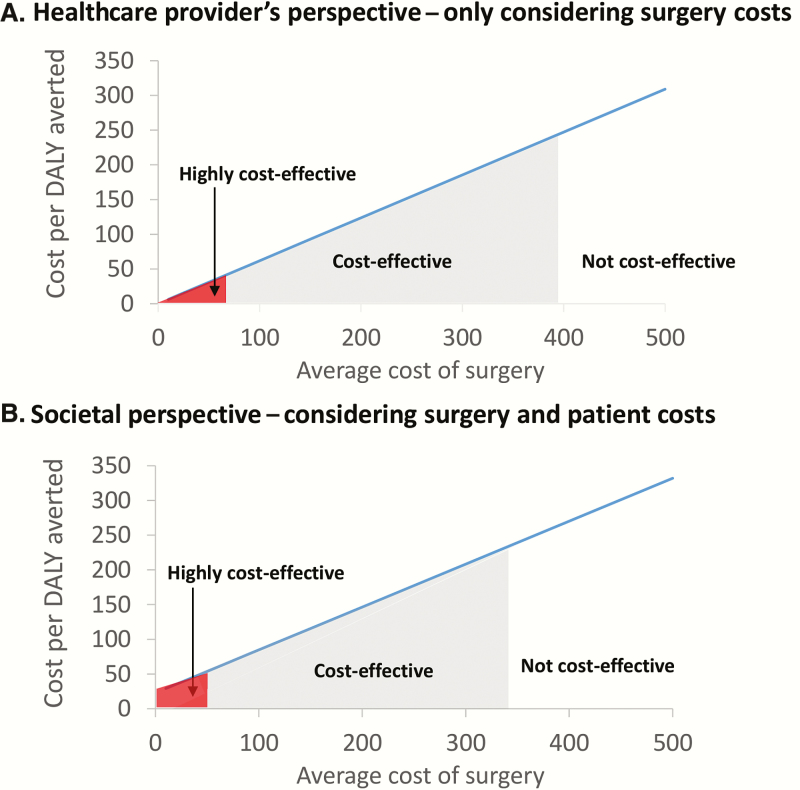
Sensitivity of the cost-effectiveness of hydrocelectomy to the assumed average surgery cost. *A*, Only the cost of the surgery is considered. *B*, Other costs that are incurred by patients, such as lost wages, food, and transportation, are also considered. Note that in some settings, patients have to pay for the surgery. This would change the cost-effectiveness under the healthcare provider’s perspective (but not the societal perspective, as this includes the costs incurred by the patients). The assumptions and baseline parameters are outlined in Supplementary Tables S6 and S7. If the cost-effectiveness ratio was less than $246 per disability-adjusted life years (DALY) averted, it was considered cost-effective and highly cost-effective if less than $41 per DALY. This is based on the thresholds established by the World Bank for low income countries [15] (inflated to their 2014 equivalent [11]). Abbreviation: DALY, disability-adjusted life years.

Under the healthcare provider’s perspective, we projected that in a low income country a hydrocelectomy would be classed as highly cost-effective if the surgery cost less than $66 and classed as cost-effective if less than $398 (Figure 3). The cost-effectiveness slightly decreased when we used the wider societal perspective for the costs, which includes the costs incurred by patients (Figure 3). However, we still found that it would be clearly classed as cost-effective for a wide range of surgery costs.

#### Sensitivity Analysis

Table 3 illustrates how sensitive the estimated cost-effectiveness of hydrocelectomy is to a range of parameters. The results were most sensitive to the assumed average age of patients at the time of surgery and to life expectancy, which determine the number of years of averted morbidity. However, even when we used the upper range of these parameters, the finding that the surgery was cost-effective was robust for the range of costs reported in the literature.

**Table 3.** Sensitivity of the Cost Required for a Hydrocelectomy to Be Classed as Cost-Effective in a Low Income Country

**Parameter and Range Investigated**	**Cost Threshold Under Which Surgery Is Classed as Cost-Effective (less then $246 per DALY averted**)	**Cost Threshold Under Which Surgery Is Classed as Highly Cost-Effective (less then $41 per DALY averted**)
Baseline Parameters	$398	$66
Average age of patients undergoing hydrocele surgery (25- to 50-year-olds)	$246–$561	$44–$93
Average life expectancy in lymphatic filariasis–endemic areas (55- to 75-year olds)	$264–$511	$44–$85
Average success rate of the surgery (60%–98%)	$275–$448	$46–$75
Lag of the health benefit after surgery (0–6 months)	$389–$400	$65–$67
Average reduction in hydrocele-related morbidity due to surgery (60%–98%)	$265–$433	$44–$72
Discount rate (0%–6%)	$328–$528	$55–$88
DALY weight (0.073–0.157)	$264–$568	$44–$95

The assumptions and baseline parameters are outlined in Supplementary Table S6. The success rate includes surgeries that did not reverse the condition or resulted in the patient having severe adverse outcomes. Results assume the healthcare provider’s perspective. The cost-effectiveness thresholds are based on those established by the World Bank for low income countries [15] (inflated to their 2014 equivalent [11]). Higher cost-effectiveness thresholds would be applicable for middle-income countries.

Abbreviation: DALY, disability-adjusted life years.

## DISCUSSION

### Preventive Chemotherapy

The preventive chemotherapy provided under the GPELF between 2000–2014 was found to be very cost-effective, with a cost per DALY averted comparable to the top 10 most cost-effective interventions reported in the second edition of the Disease Control Priorities in Developing Countries (DCP2) project, even when the economic value of the donated drugs was included. The preventive chemotherapy was also found to have a very high benefit-cost ratio, indicating that it is a very good investment in public health (Table 2).

As the time horizon was only over the lifetime of those treated between 2000 and 2014, these estimates only indicate the cost-effectiveness/benefit of the program’s long-term impact on that specific group; no projections were made for the expansion of MDA programs after 2014 and their resulting benefits. However, the program’s significant long-term health and economic benefits [8] could be diminished if the MDA programs are not continued until elimination is achieved and infection was allowed to resurge to precontrol levels in these treated populations.

The estimated cost-effectiveness and cost-benefit of the GPELF were lower when the economic value of the donated drugs was included in the analysis, particularly for African countries that use the ivermectin and albendazole regimen due to the higher reported economic value of ivermectin [16]. When we looked only at countries that use the ivermectin and albendazole regimen, it was found that the preventive chemotherapy would no longer be classed as cost-effective when using the World Bank thresholds (although only marginally), but it remained highly cost-effective based on the WHO thresholds [17]. Despite this result, the treatment program would still be considered cost-effective as a whole/worldwide. It should be noted that it is difficult to estimate the true economic value of the donated drugs, and there is variation in the values used. Furthermore, it is important to remember that the foundation of the GPELF is based on the long-term and sustained commitment of drug donations for as long as needed until LF is eliminated [18]. Because of this, governments/donors will never have to finance the cost of drugs. Consequently, understanding the cost-effectiveness/cost-benefit when the value of the donated drugs is excluded is more relevant for policy- and decision-making.

#### Limitations

The limitations of the models to estimate the impact of preventive chemotherapy have been described elsewhere [8]. Of particular note, the DALY calculations do not account for any excess mortality resulting from clinical LF and, as in the Global Burden of Disease Study (2010), we did not quantify any disability resulting from mental illness and depression in clinical disease patients and their caregivers, which has been shown to be significant [19]. Furthermore, the reductions in the at-risk population due to treatment were not estimated with a dynamic transmission model. However, the large health impact of preventive chemotherapy was found to be robust to a wide range of assumptions within the univariate sensitivity analysis [8]. A more advanced multivariate sensitivity analysis would be ideal but would require more data than are available.

Of particular note, our estimates were sensitive to the assumed average treatment delivery cost, which is surrounded by a degree of uncertainty. However, our findings were robust even when we used the costs above the confidence interval estimated by the costing model [9, 10]. Furthermore, in some settings volunteers distribute the drugs (likely resulting in lower delivery costs than assumed in this analysis). It should be noted that the unit delivery costs for programs may become considerably higher as they approach the “last mile” toward elimination. For example, intervention costs will likely be higher in areas coendemic with both Loa loa and river blindness [20]. In addition, when looking at the economic benefit of achieving elimination (beyond the scope of this study), it will be important to consider the post-MDA surveillance costs [21]. However, in this context the benefits would be occurring over a longer time frame than considered in this analysis and would therefore be larger.

The majority of economic benefits included in the cost-benefit calculations resulted from prevented income loss, which was calculated using the human capital approach. However, the precise benefit to the economy as a whole is very difficult to calculate, and the estimates do not account for the potential for labor to be replaced by other noninfected individuals. Therefore, these cost-benefit ratios should be interpreted with a degree of caution. However, it is still meaningful to compare them to estimates reported for other diseases estimated using the same approach.

### Hydrocele Surgery and Morbidity Management

We projected that hydrocelectomies are typically very cost-effective. Currently, only about one-third of national programs have reported activities to manage morbidity and prevent disability in people suffering from LF [6]. These results give a strong indication that hydrocele surgeries are cost-effective and further support the importance of efforts to encourage and assist all endemic countries to build capacity in this area. This highlights the need for further research in this area so that more cost and effectiveness data can be collected and more advanced analyses conducted.

#### Limitations

An important limitation of this preliminary analysis is that very few studies have reported the rates of complications and recurrence following LF-related hydrocelectomy [3], and there is little data that is not anecdotal in nature regarding the degree and time frame to which hydrocelectomy improves morbidity/productivity [14]. Furthermore, there is an absence of cost data and therefore little understanding regarding how the costs vary in different settings. The absence of data on outcomes and costs makes it impossible to compare the cost-effectiveness of one surgical technique to another. However, it should be noted that the overall conclusion that hydrocelectomy is cost-effective was robust to a wide range of assumptions regarding its costs and effectiveness.

It is also important to highlight that lymphedema management is an important component of LF morbidity management programs. Due to the absence of data regarding the different methods used, their cost, clinical effectiveness, and how variable these are across different settings, it was not possible for us to perform a cost-effectiveness analysis of these programs. However, it is noteworthy that Stillwaggon et al [22] recently estimated that the economic benefits over a patient’s lifetime resulting from a lymphedema management program in India were more than 130 times its costs, highlighting how cost-effective these programs can be.

To allow for more thorough cost-effectiveness analyses of LF morbidity management strategies (across a range of settings), more data relating to their costs, resource requirements, clinical effectiveness, and incidence of complications/relapse for the different potential techniques/methods are urgently needed.

It should be noted that we are modeling the impact and cost of a typical hydrocelectomy/case of hydrocele. For more extreme clinical cases, a hydrocelectomy may be less likely to be successful or more likely to result in complications, and individuals may require more post-operative care. While surgeries for more extreme clinical cases are likely to be more expensive, the corresponding benefit and gain for the patient would also be larger.

### Further Considerations

It is important to note that the GPELF would have a positive effect on the quality of life for clinical patients and their families, a benefit that is not captured by the explored effectiveness metrics. For example, the stigma associated with the clinical disease can prevent patients from playing a full role in society, often reducing marital prospects. This can result in adverse social and economic repercussions, not only for the patient but also for their family. The GPELF uses broad-spectrum antiparasitic drugs and, consequently, it has substantial ancillary benefits on other parasitic diseases (described in more detail in [2, 8]).

Though the analysis shows that the current preventive chemotherapy strategy is cost-effective, that does not mean that alternative strategies should not be investigated to help reach the 2020 goals, such as the use of triple-drug therapy [23] and interventions that can be used in Loa loa coendemic areas.

Within this study, we used the cost-effectiveness thresholds established by the World Bank for low income countries, which are more conservative than the WHO-CHOICE thresholds [15, 17]. There is debate and uncertainty surrounding the definition of the most appropriate thresholds [24, 25]. A review of different approaches is presented by Marseille et al and Shillcutt et al [25, 26]. It should be noted that higher cost-effectiveness thresholds would be applicable for middle-income countries.

This work further highlights that NTD control programs can be very cost-effective [27, 28]. Despite this, funding for NTD control is often disproportionately low, which does not reflect the respective importance of these diseases [29].

## CONCLUSIONS

Both the preventive chemotherapy and hydrocele surgery provided under the GPELF were estimated to be incredibly cost-effective and offer a very good investment in public health. Due to the limitations of any such analysis, there is some uncertainty surrounding these estimates. However, the overall findings were very robust within the sensitivity analysis. It is important to note that the program’s large health and economic impacts would be diminished if the LF programs were not continued until elimination is achieved. The work highlights the value of the GPELF and the vital need for further investment to allow it to continue.

## Supplementary Material

Supplementary Data

## Notes

***Financial support.*** H. C. T. and A. A. B. are supported by the London Centre for Neglected Tropical Disease Research (funded by GlaxoSmithKline) HCT is supported by the Wellcome Trust [089276/B/09/7].

***Potential conflicts of interest.*** M. H. B. currently works for GlaxoSmithKline. All other authors certify no potential conflicts of interest. All authors have submitted the ICMJE Form for Disclosure of Potential Conflicts of Interest. Conflicts that the editors consider relevant to the content of the manuscript have been disclosed.
