# Supplementary material for: Investment Success in Public Health: An Analysis of the Cost-Effectiveness and Cost-Benefit of the Global Programme to Eliminate Lymphatic Filariasis
Source: Clin Infect Dis. 2016 Dec 10;64(6):728–35. doi: 10.1093/cid/ciw835 (PMC5404931; doi:10.1093/cid/ciw835)
Supplement: Supplementary Data [file ciw835_Supplementary_Data.zip › GPELF_cost_benefit_Supporting_Infomation_CID_revisions.docx]

**Supporting Information**

**Delivery costs**

The delivery costs of MDA were estimated using a recently developed web-based regression model (<https://healthy.shinyapps.io/benchmark>) developed by the WHO [[1](#_ENREF_1), [2](#_ENREF_2)]. The model was based on a systematic review (identifying 34 eligible studies) and a meta-regression of the obtained cost data and estimates the delivery cost per treatment (with 95% CI) based on the number treated and the local GDP [[1](#_ENREF_1)]. Using treatment number data from the PCT databank [[3](#_ENREF_3)] and the GDP for relevant endemic countries [[4](#_ENREF_4)], we used the model to estimate country-specific financial and economic unit delivery costs (with 95% CI) for each year of the GPELF (2000-2014) (Table 2). Crucially, the model accounts for the economies of scale associated with MDA (i.e. the reduction in the delivery cost per treatment as the number treated is increased). Consequently, the delivery cost per treatment for each country changes over time. To be conservative, we capped the number treated parameter within the model at three million. This was done to avoid over-extrapolating the cost data used to parameterise the model, and overestimating the economies of scale (i.e. we assumed that the delivery cost per treatment does not decrease further when treating over three million people in an MDA round). In addition, we used the model parametrisation relating to the use of paid health workers and not community volunteers for the drug distribution (resulting in a higher unit delivery cost). The parameter values used are described in Supporting Table S5. The model outputs costs were deflated from 2015 to 2014 US$ [[5](#_ENREF_5)].

| **Supporting Table S1: Summary of the MDA treatments provided by GPELF (2000-2014)** | | | | |
| --- | --- | --- | --- | --- |
| **WHO Region** | **Pre-control number at risk of infection *(millions)***^†^ | **Treatments with ALB & IVM *(millions)***^†^ | **Treatments with**  **ALB & DEC or DEC alone *(millions)***^†^ | **Total number of treatments**  ***(millions)*^†^** |
| **AMRO** | 14 | 0 | 47 | 47 |
| **AFRO** | 425 | 830 | 47 | 877 |
| **EMRO** | 23 | 0.6 | 15 | 15.6 |
| **WPRO** | 45 | 0 | 193 | 193 |
| **SEARO** | 902 | 0 | 4,494 | 4,494 |
| **All Regions** | **1,409** | **830** | **4,797** | **562** |
| *AMRO: Region of the Americas, AFRO: African Region, EMRO: Eastern Mediterranean Region, WPRO: Western Pacific Region, SEARO: South-East Asia Region, Albendazole: ALB*, *Diethylcarbamazine: DEC, Ivermectin: IVM*  *† Data taken from [*[*3*](#_ENREF_3)*,* [*6*](#_ENREF_6)*].* | | | | |

| **Supporting Table S2: Benefit for individuals and the health systems over lifetime of the benefit cohorts** | | | | | |
| --- | --- | --- | --- | --- | --- |
| **Benefit cohort size *(millions)*** | **DALYs**  **averted* *(millions)*** | **Direct costs for individuals prevented - medical expenses *(billions)*** | **Direct costs for the health system** **prevented *(billions)*** | **Indirect costs for individuals prevented - lost wages *(billions)*** | **Total *(billions)*** |
| 46 | 115 (76-164) | 3 | 3.5 | 94 | 100.5 |
| * *Range based on the 95% uncertainty interval of the disability weight (0.073–0.157). No distinction in the weight was made between hydrocele and lymphedema and age-weighting was not applied [*[*7*](#_ENREF_7)*]. It should be noted that unlike the results in [*[*8*](#_ENREF_8)*], the DALYs presented here have been discounted into the future (at 3% per year [*[*9*](#_ENREF_9)*])* – *common practice when performing an economic evaluation [*[*9*](#_ENREF_9)*].The indirect cost estimates were calculated using the human capital approach. A combination of four wage sources was used to estimate the fair market value of time for an agricultural worker with LF infection (to ensure a conservative estimate, the lowest wage value was used).Results are taken from [*[*8*](#_ENREF_8)*]. Costs are expressed in US$ 2014 prices.* | | | | | |

| **Supporting** **Table S3: Summary of the sensitivity analysis performed on the effectiveness of preventive chemotherapy (based on [**[**8**](#_ENREF_8)**]).** | | | | |
| --- | --- | --- | --- | --- |
| **Parameter** | **Hydrocele average estimate** | | **Lymphedema average estimate** | **Sources** |
| ***Disease Progression & Incidence Rates*** |  | |  |  |
| Percentage of clinical patients who experience ADL episodes per year | 70% (45-90%) | | 95% (90-95%) | [[10-18](#_ENREF_10)] |
| Frequency of ADL episodes for clinical patients (in absence of MDA) | 2 (0-7) per year | | 4 (0-7) per year | [[10-18](#_ENREF_10)] |
| Average duration of an ADL episode | 4 (1-9) days | | 4 (1-9) days | [[10-18](#_ENREF_10)] |
| Disability weight | 0.11 (0.073–0.157) | | 0.11 (0.073–0.157) | [[7](#_ENREF_7)] |
| Mean age of the benefit cohorts (years) | Cohort 1: 20 (30)  Cohort 2: 20 (30)  Cohort 3: 30 (40) | | Cohort 1: 20 (30)  Cohort 2: 20 (30)  Cohort 3: 30 (40) |  |
| ***Patient Medical Expenses and Treatment-Seeking Behaviour*** | | |  |  |
| Percentage of patients with ADL seeking treatment per episode | | 55% (55-70%)  India 70% (70%-98%) | 55% (55-70%)  (India 75% (75%-98%)) | [[10](#_ENREF_10), [11](#_ENREF_11), [15](#_ENREF_15), [19](#_ENREF_19), [20](#_ENREF_20)] |
| Percentage of chronic disease patients seeking treatment | | 20% (20-50%)  India: 50% (41-80%) | 30% (30-55%)  India 55% (48-100%) | [[19](#_ENREF_19), [21](#_ENREF_21), [22](#_ENREF_22)] |
| Average patient medical expenses per ADL episode | | +- 20% of baseline value | +- 20% of baseline value | [[10](#_ENREF_10), [11](#_ENREF_11), [19-21](#_ENREF_19), [23](#_ENREF_23), [24](#_ENREF_24)] |
| Average patient medical expenses for chronic disease per year | | +-20% of baseline value | +-20% of baseline value | [[10](#_ENREF_10), [11](#_ENREF_11), [19-21](#_ENREF_19), [23](#_ENREF_23), [24](#_ENREF_24)] |
| ***Lost Productivity & Wages*** | |  |  |  |
| Work days per year | | 300 (261-365) days | 300 (261-365) days |  |
| Percentage of work hours lost per day during an ADL episode | | 75% (50–93%) | 75% (50–93%) | [[13](#_ENREF_13), [15](#_ENREF_15), [17](#_ENREF_17), [25](#_ENREF_25)] |
| Percentage of work hours lost due to chronic disease | | 15% (9–24%) | 19% (11–31%) | [[13](#_ENREF_13), [20](#_ENREF_20), [21](#_ENREF_21), [26](#_ENREF_26)] |
| ***Discounting*** | |  |  |  |
| Discount rate | | 3% (0-6%) | 3% (0-6%) | [[9](#_ENREF_9)] |
| ***Impact of Treatment*** | |  |  |  |
| The reduction in transmission experienced by the treated population | | Year 1: 50% (35%)  Year 2: 75% (53%)  Year 3: 88% (62%)  Year 4: 94% (66%)  Year 5 95% (67%) | Year 1: 50% (35%)  Year 2: 75% (53%)  Year 3: 88% (62%)  Year 4: 94% (66%)  Year 5 95% (67%) | [[27](#_ENREF_27)] |
| Reduction in the frequency of ADL episodes by MDA | | 50% (15-88%) | 50% (15-88%) | [[28-30](#_ENREF_28)]. |
| Percentage of chronic disease alleviated by MDA | | 10% (0-90%) | 15% (0-69%) | [[28](#_ENREF_28), [31-36](#_ENREF_31)] |
| *Based on [*[*37*](#_ENREF_37)*], though updated where appropriate. ADL:acute adenolymphangitis. MDA: mass drug administration.* | | | | |

| **Supporting Table S4: Drug costs and their economic value.** | | | | |  |
| --- | --- | --- | --- | --- | --- |
| **Drug and dose** | **Average number of tablets needed per treatment*** | **Cost/value of each tablet** | **Shipping cost per tablet** | **Average cost/value per treatment**† | **Cost type** |
| DEC  (100 mg per tablet) | 2.75 [[38](#_ENREF_38)] | US$0.0144 per tablet ^a^ | Included in the tablet cost estimate | US$0.04356 per treatment | Financial  (2000-2012)  Mixed‡  (2013-2014) |
| Albendazole  (400 mg per tablet) | 1 [[39](#_ENREF_39)] | US$0.045 per tablet ^b^ [[40](#_ENREF_40)] | US$0.0019 [[41](#_ENREF_41)] | US$0.0516 per treatment | Economic (2000-2014) |
| Ivermectin  (3 mg per tablet) | 2.8 [[42](#_ENREF_42)] | US$1.5 per tablet ^c^ [[42](#_ENREF_42)] | US$0.005^c^ [[42](#_ENREF_42)] | US$4.635 per treatment | Economic (2000-2014) |
| ** For DEC and ivermectin the number of required tablets depends on the size of the individual and therefore the overall average is not a whole number.*  *† Includes a wastage factor of 10%.*  *^a^ Eisai, Unpublished. ^b^* *GSK, Unpublished. ^c^ Mectizan Donation Program, Unpublished.*  *It should be noted that these are the costs/values reported by the drugs companies that donate them. However, it is possible to procure the drugs at lower prices (see International Drug Price Indicator Guide (http://erc.msh.org/priceguide)). Albendazole: GlaxoSmithKline changed the valuation of the donated albendazole in late 2008 (published in 2009) to U.S. $0.045 per tablet (from $0.19 per tablet) ^b^[*[*40*](#_ENREF_40)*].*  *‡ During this time period, this was a mixture of financial and economic costs. In 2013, Sanofi assisted the WHO with a donation of 130 million tablets and the remaining tablets were purchased. From October 2013 EISAI donated DEC to all requesting countries.* | | | | | |

| **Supporting Table S5: MDA costing model parameters [**[**1**](#_ENREF_1)**,** [**2**](#_ENREF_2)**]** | |
| --- | --- |
| **Model parameter** | **Value** |
| Number treated | Taken from the WHO’s PCT databank [[3](#_ENREF_3)] |
| Coverage | 85%* |
| National or subnational | National |
| School-based delivery; Yes/No | No |
| Number of rounds per year | 1 |
| Number of diseases integrated | 1 |
| Year of implementation | Varied |
| GDP per capita (2015 US$) | Taken from [[4](#_ENREF_4)] |
| Population density (people per square km) | 134* |
| Random or fixed effects | Random |
| * *The average (median) observation of the studies used to parameterise the costing model [*[*1*](#_ENREF_1)*,* [*2*](#_ENREF_2)*].* | |

| **Supporting Table S6: Summary of the sensitivity analysis for hydrocele surgery** | | |
| --- | --- | --- |
| **Parameter** | **Baseline value [range]** | **Reference** |
| Average age of patients undergoing hydrocele surgery | 40 years [25-50 year olds] | ***^‡^*** |
| Average life expectancy in LF endemic areas | 65 years [55-75 years] | [[43](#_ENREF_43)] |
| Average success rate of the surgery | 87% [60-98%]* | [[44](#_ENREF_44)] |
| The lag of the health benefit after surgery | 1 month [0-6 months] | [[45](#_ENREF_45)]***^‡^*** |
| Average reduction in hydrocele related morbidity due to surgery | 90% [60-98%] | ***^‡^*** |
| Discount rate | 3% [0-6%] | [[9](#_ENREF_9)] |
| DALY weight | 0.11 [0.073–0.157]^†^ | [[7](#_ENREF_7)] |
| *‡Based on the expert opinion of Dr. Sunny Mante.*  *** *We assumed that a typical hydrocelectomy has a success rate of 87% (with the remaining 13% either failing to reverse the condition, or resulting in the patient having severe adverse outcomes.*  *† As we assumed only a percentage of hydrocele related morbidity was being alleviated, the DALY weight was reduced proportionally within the benefit calculations.* | | |

| **Supporting Table S7: Summary of the additional costs incurred by patients for a hydrocelectomy** | | |
| --- | --- | --- |
| **Cost type** | **Value** | **Reference** |
| Food and transportation | US$25 | [[45](#_ENREF_45)] |
| Lost wages | US$12 | [[8](#_ENREF_8), [45](#_ENREF_45)] |
| *Ahorlu et al. [*[*45*](#_ENREF_45)*] estimated that patients spend US$20–30 for food and transportation to the hospital, and spend an average of 4–12 days in the hospital (and therefore on average lose eight working days income). To quantify the lost wages we applied our estimated weighted average minimum wage of the GPELF countries (US$1.50 [*[*8*](#_ENREF_8)*]) to the average number of lost working days.* | | |


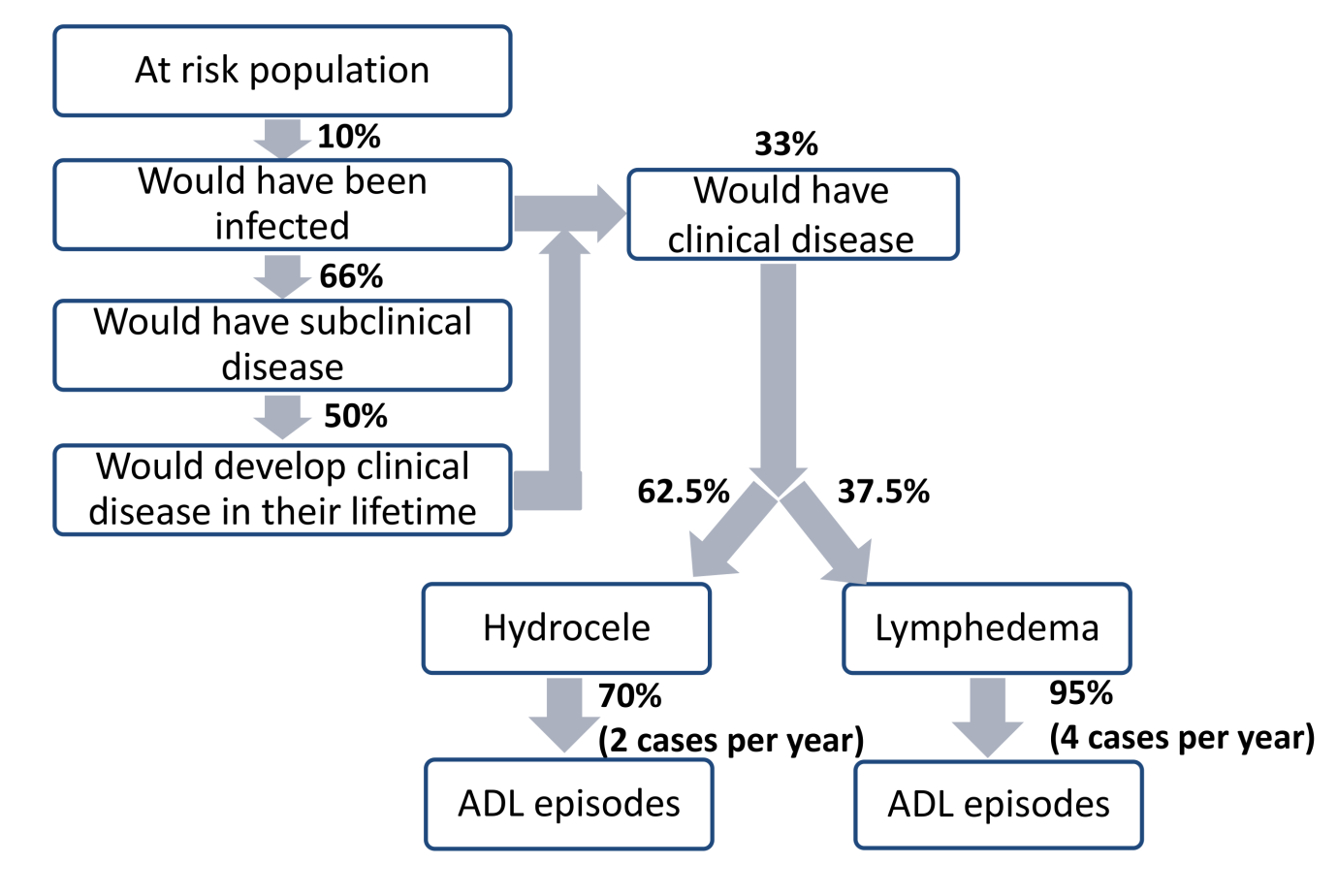
**Figure S1: Baseline model assumptions.** *Assumptions based on [*[*37*](#_ENREF_37)*,* [*46*](#_ENREF_46)*]. The sources for the parameters are outlined in Table 2. Modified from* [[8](#_ENREF_8)].


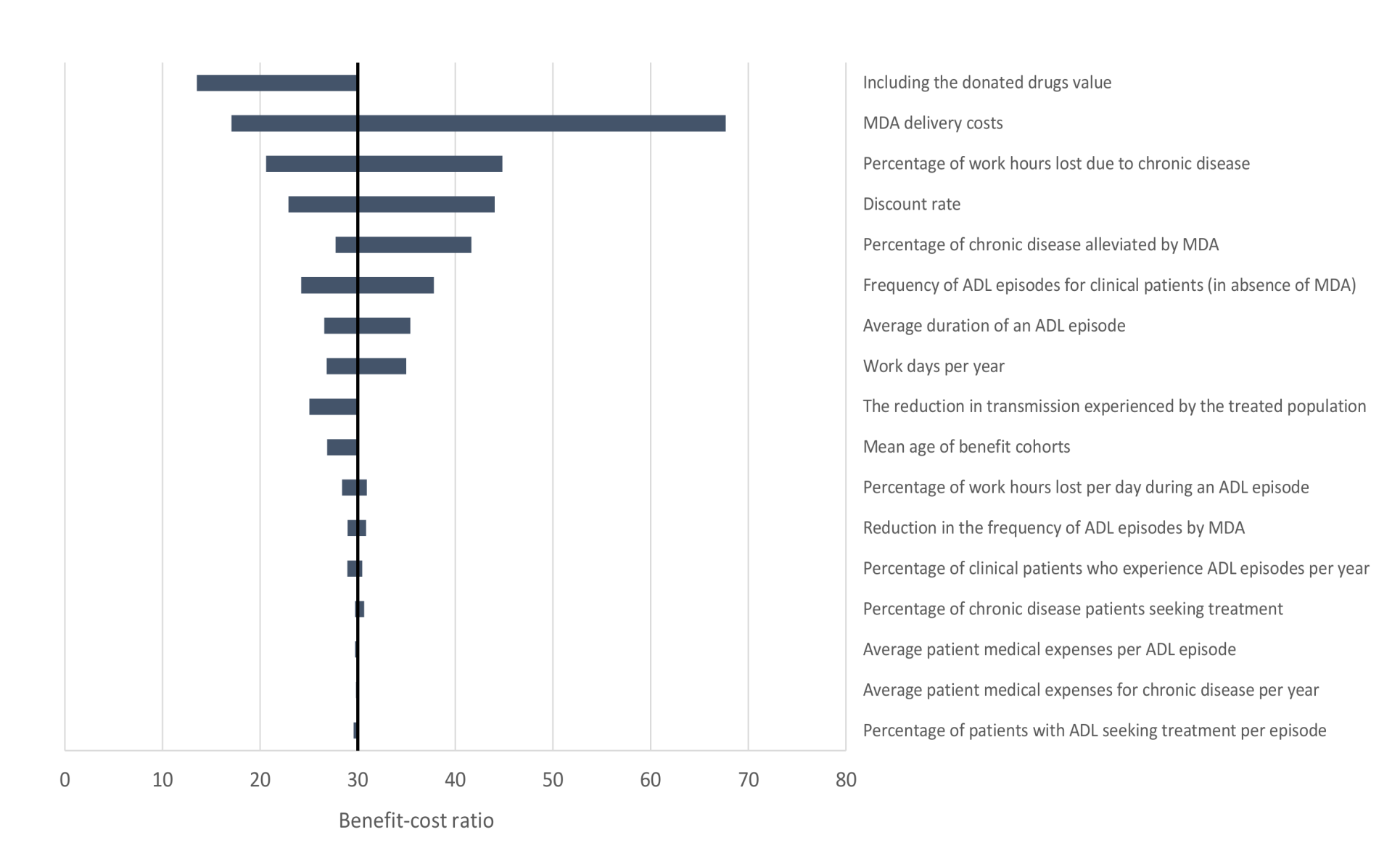
**Supporting** **Figure S2: Tornado plot illustrating the impact of the sensitivity analysis on the estimated cost-effectiveness and cost-benefit of the GPELF (2000-2014).** *The parameter ranges investigated are shown in Supporting Table S3 and Table 2. Baseline results use the economic delivery costs (excluding the donated drugs value)*.

**References**

1. Fitzpatrick C, Madin-Warburton M, Schneiderd T, et al. Benchmarks for the cost per person of mass treatment against neglected tropical diseases: a literature review and metaregression with web-based software application. PLoS Negl Trop Dis **2016**; Minor revisions.

2. Benchmarks for the cost per person of mass treatment against neglected tropical diseases. Available at: https://healthy.shinyapps.io/benchmark.

3. World Health Organisation. PCT databank: Lymphatic filariasis. Available at: <http://www.who.int/neglected_diseases/preventive_chemotherapy/lf/en/>.

4. World Bank. GDP per capita (constant 2005 US$) Available at: <http://data.worldbank.org/indicator/NY.GDP.PCAP.KD?page=2>.

5. International monetary fund. Available at: https://[www.imf.org/external/pubs/ft/weo/2015/01/weodata/weoselgr.aspx](http://www.imf.org/external/pubs/ft/weo/2015/01/weodata/weoselgr.aspx).

6. World Health Organization. 489 Global programme to eliminate lymphatic filariasis: progress report, 2014. Wkly Epidemiol Rec **2015**; 90(38): 489-504.

7. Salomon JA, Vos T, Hogan DR, et al. Common values in assessing health outcomes from disease and injury: disability weights measurement study for the Global Burden of Disease Study 2010. Lancet **2012**; 380(9859): 2129-43.

8. Turner HC, Bettis, A.A., Chu, B.K., McFarland, D.A., Hooper, P.J., Ottesen, E.A. and Bradley, M.H. . The Health and Economic Benefits of the Global Programme to Eliminate Lymphatic Filariasis (2000–2014). Infect Dis Poverty **2016**; 5(1): 54.

9. WHO-CHOICE. Making choices in health: WHO guide to cost-effectiveness analysis. Geneva: World Health Organization, **2003**.

10. Krishnamoorthy K. Estimated costs of acute adenolymphangitis to patients with chronic manifestations of bancroftian filariasis in India. Indian J Public Health **1999**; 43(2): 58-63.

11. Ramaiah KD, Ramu K, Kumar KN, Guyatt H. Epidemiology of acute filarial episodes caused by Wuchereria bancrofti infection in two rural villages in Tamil, Nadu, south India. Trans R Soc Trop Med Hyg **1996**; 90(6): 639-43.

12. Pani SP, Yuvaraj J, Vanamail P, et al. Episodic adenolymphangitis and lymphoedema in patients with bancroftian filariasis. Trans R Soc Trop Med Hyg **1995**; 89(1): 72-4.

13. Ramaiah KD, Radhamani MP, John KR, et al. The impact of lymphatic filariasis on labour inputs in southern India: results of a multi-site study. Ann Trop Med Parasitol **2000**; 94(4): 353-64.

14. Babu BV, Nayak AN, Dhal K. Epidemiology of episodic adenolymphangitis: a longitudinal prospective surveillance among a rural community endemic for bancroftian filariasis in coastal Orissa, India. BMC Public Health **2005**; 5: 50.

15. Gyapong JO, Gyapong M, Adjei S. The epidemiology of acute adenolymphangitis due to lymphatic filariasis in northern Ghana. Am J Trop Med Hyg **1996**; 54(6): 591-5.

16. Kessel JF. Disabling effects and control of filariasis. Am J Trop Med Hyg **1957**; 6(3): 402-14; discussion, 15.

17. Gasarasi DB, Premji ZG, Mujinja PG, Mpembeni R. Acute adenolymphangitis due to bancroftian filariasis in Rufiji district, south east Tanzania. Acta Trop **2000**; 75(1): 19-28.

18. Sabesan S, Krishnamoorthy K, Pani SP, Panicker KN. Mandays lost dueto repeated attacks of lymphatic filariasis. Trends in Life Sciences **1992**; 7: 5-7.

19. Nanda B, Krishnamoorthy K. Treatment seeking behaviour and costs due to acute and chronic forms of lymphatic filariasis in urban areas in south India. Trop Med Int Health **2003**; 8(1): 56-9.

20. Babu BV, Nayak AN, Dhal K, Acharya AS, Jangid PK, Mallick G. The economic loss due to treatment costs and work loss to individuals with chronic lymphatic filariasis in rural communities of Orissa, India. Acta Trop **2002**; 82(1): 31-8.

21. Ramaiah KD, Guyatt H, Ramu K, Vanamail P, Pani SP, Das PK. Treatment costs and loss of work time to individuals with chronic lymphatic filariasis in rural communities in south India. Trop Med Int Health **1999**; 4(1): 19-25.

22. Gyapong JO, Gyapong M, Evans DB, Aikins MK, Adjei S. The economic burden of lymphatic filariasis in northern Ghana. Ann Trop Med Parasitol **1996**; 90(1): 39-48.

23. Management Science for Health. International Drug price Indicator Guide. Available at: <http://erc.msh.org/mainpage.cfm?file=1.0.htm&module=DMP&language=English>.

24. Health Action International. Database of medicine prices, availability, affordability and price components. Available at: <http://www.haiweb.org/MedPriceDatabase/>.

25. Ramaiah KD, Ramu K, Guyatt H, Kumar KN, Pani SP. Direct and indirect costs of the acute form of lymphatic filariasis to households in rural areas of Tamil Nadu, south India. Trop Med Int Health **1998**; 3(2): 108-15.

26. Addiss DG, Brady MA. Morbidity management in the Global Programme to Eliminate Lymphatic Filariasis: a review of the scientific literature. Filaria J **2007**; 6: 2.

27. Hooper PJ, Chu BK, Mikhailov A, Ottesen EA, Bradley M. Assessing progress in reducing the at-risk population after 13 years of the global programme to eliminate lymphatic filariasis. PLoS Negl Trop Dis **2014**; 8(11): e3333.

28. Ciferri F, Siliga N, Long G, Kessel JF. A filariasis-control program in American Samoa. Am J Trop Med Hyg **1969**; 18(3): 369-78.

29. Fan PC, Peng HW, Chen CC. Follow-up investigations on clinical manifestations after filariasis eradication by diethylcarbamazine medicated common salt on Kinmen (Quemoy) Islands, Republic of China. J Trop Med Hyg **1995**; 98(6): 461-4.

30. Das L, Subramanyam Reddy G, Pani S. Some observations on the effect of Daflon (micronized purified flavonoid fraction of Rutaceae aurantiae) in bancroftian filarial lymphoedema. Filaria J **2003**; 2(1): 5.

31. Bockarie MJ, Tisch DJ, Kastens W, et al. Mass treatment to eliminate filariasis in Papua New Guinea. N Engl J Med **2002**; 347(23): 1841-8.

32. Partono F. Treatment of elephantiasis in a community with timorian filariasis. Trans R Soc Trop Med Hyg **1985**; 79(1): 44-6.

33. Partono F, Purnomo, Oemijati S, Soewarta A. The long term effects of repeated diethylcarbamazine administration with special reference to microfilaraemia and elephantiasis. Acta Trop **1981**; 38(3): 217-25.

34. Meyrowitsch DW, Simonsen PE, Makunde WH. Mass diethylcarbamazine chemotherapy for control of bancroftian filariasis through community participation: comparative efficacy of a low monthly dose and medicated salt. Trans R Soc Trop Med Hyg **1996**; 90(1): 74-9.

35. Mackenzie CD, Lazarus WM, Mwakitalu ME, Mwingira U, Malecela MN. Lymphatic filariasis: patients and the global elimination programme. Ann Trop Med Parasitol **2009**; 103 Suppl 1: S41-51.

36. March HN, Laigret J, Kessel JF, Bambridge B. Reduction in the prevalence of clinical filariasis in Tahiti following adoption of a control program. Am J Trop Med Hyg **1960**; 9: 180-4.

37. Chu BK, Hooper PJ, Bradley MH, McFarland DA, Ottesen EA. The economic benefits resulting from the first 8 years of the Global Programme to Eliminate Lymphatic Filariasis (2000-2007). PLoS Negl Trop Dis **2010**; 4(6): e708.

38. Stolk WA, ten Bosch QA, de Vlas SJ, Fischer PU, Weil GJ, Goldman AS. Modeling the Impact and Costs of Semiannual Mass Drug Administration for Accelerated Elimination of Lymphatic Filariasis. PLoS Negl Trop Dis **2013**; 7(1): e1984.

39. World Health Organization. Preventive chemotherapy in human helminthiasis: coordinated use of anthelminthic drugs in control interventions: a manual for health professionals and programme managers. Geneva: WHO, **2006**.

40. Goldman AS, Brady MA, Direny A, et al. Costs of Integrated Mass Drug Administration for Neglected Tropical Diseases in Haiti. The American Journal of Tropical Medicine and Hygiene **2011**; 85(5): 826-33.

41. Goldman AS, Guisinger VH, Aikins M, et al. National mass drug administration costs for lymphatic filariasis elimination. PLoS Negl Trop Dis **2007**; 1(1): e67.

42. Coffeng LE, Stolk WA, Zouré HGM, et al. African Programme for Onchocerciasis Control 1995–2015: Model-Estimated Health Impact and Cost. PLoS Negl Trop Dis **2013**; 7(1): e2032.

43. World Health Organisation. Global Health Observatory data repository. Available at: <http://apps.who.int/gho/data/view.main.60280?lang=en>.

44. Kiddoo DA, Wollin TA, Mador DR. A population based assessment of complications following outpatient hydrocelectomy and spermatocelectomy. J Urol **2004**; 171(2 Pt 1): 746-8.

45. Ahorlu CK, Dunyo SK, Asamoah G, Simonsen PE. Consequences of hydrocele and the benefits of hydrocelectomy: a qualitative study in lymphatic filariasis endemic communities on the coast of Ghana. Acta Trop **2001**; 80(3): 215-21.

46. Ottesen EA, Hooper PJ, Bradley M, Biswas G. The global programme to eliminate lymphatic filariasis: health impact after 8 years. PLoS Negl Trop Dis **2008**; 2(10): e317.
